# Reassessment of the Conservation Status of the Endemic and Endangered Plant *Dalbergia odorifera* T.C. Chen in Hainan, China

**DOI:** 10.1002/ece3.72465

**Published:** 2025-11-09

**Authors:** Chumin Ye, Kai Zhang, Xinli Gui, Yukai Chen, Haifu Meng

**Affiliations:** ^1^ Ministry of Education Key Laboratory for Ecology of Tropical Islands, Key Laboratory of Tropical Animal and Plant Ecology of Hainan Province, College of Life Sciences Hainan Normal University Haikou People’s Republic of China

**Keywords:** conservation, *Dalbergia odorifera*, endangerment level, endemic plant, IUCN red list

## Abstract

*Dalbergia odorifera* T.C. Chen is an endemic and endangered species in Hainan, China. This study reassessed its conservation status based on the IUCN Red List Categories and Criteria Version 3.1 and its regional guidelines, as well as evaluation indicators for the threatened degree of the tiny population of wild plants endangered in China. The results showed that according to IUCN criteria, *D. odorifera* T.C. Chen was assessed as critically endangered (CR) under Criteria A1ac and D. This designation reflects the severe population decline and the extremely small number of mature individuals—only about 30—found within a total wild population of approximately 200 in Hainan. The current CR status contrasts sharply with its previous international classification of vulnerable (VU). Based on China's evaluation index system for extremely small wild plant populations, *D. odorifera* T.C. Chen was also rated as endangered (Grade IV). The outcomes from both assessment systems are congruent, confirming a status equivalent to CR. This consensus is underpinned by the species' critically small, fragmented, and declining wild population, with the total number of mature individuals remaining dangerously low. This study provides a scientific basis for the endangered level assessment of *D. odorifera* T.C. Chen and the subsequent management and protection strategies.

## Graphical abstract

*Dalbergia odorifera* T.C. Chen is an endemic and endangered species in Hainan, China. This study reassessed its conservation status based on the IUCN Red List Categories and Criteria Version 3.1 and its regional guidelines, as well as evaluation indicatiors for threatened degree of the tiny population of wild plants endangered in China. The results showed that:According to IUCN criteria, *D. odorifera* T.C. Chen was assessed as Critically Endangered (CR) under Criteria A1ac and D. This designation reflects the severe population decline and the extremely small number of mature individuals—only about 30—found within a total wild population of approximately 200 in Hainan. The current CR status contrasts sharply with its previous international classification of Vulnerable (VU).Based on China's evaluation index system for extremely small wild plant populations, *D. odorifera* T.C. Chen was also rated as Endangered (Grade IV). The outcomes from both assessment systems are congruent, confirming a status equivalent to CR. This consensus is underpinned by the species' critically small, fragmented, and declining wild population, with the total number of mature individuals remaining dangerously low. This study provides a scientific basis for the endangered level assessment of *D. odorifera* T.C. Chen and the subsequent management and protection strategies.

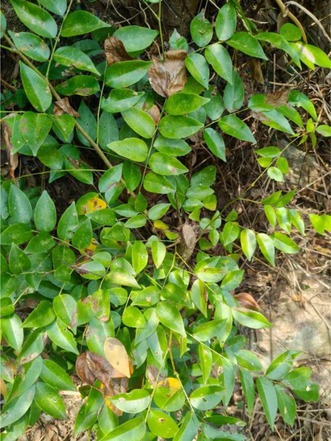

## 1 Introduction

China maintains its position as a global biodiversity hotspot, currently ranking third worldwide in floristic diversity with 36,159 documented species of vascular plants (tracheophytes) in its national botanical registry (Liu and Qin 2022; Wu et al. 2023). However, rapid demographic expansion, unregulated resource extraction, anthropogenic landscape modification, climate change, toxic substance release, non‐native species colonization, and habitat fragmentation have synergistically amplified biodiversity erosion rates. This ecological crisis trajectory has classified China among the most critically endangered (CR) nations in terms of species preservation challenges (Xiao et al. 2022). The ecological debt incurred through environmental degradation manifests most profoundly in biodiversity depletion, necessitating immediate conservation mobilization (Qin et al. 2017). As keystone components of biological diversity (Cheng and Zang 2004), rare species protection constitutes the strategic nexus of ecosystem safeguarding, whereas science‐based extinction risk appraisal establishes the operational baseline for designing mitigation frameworks (Wang et al. 2017). Standardized threat categorization provides the decision matrix for conservation prioritization (Qin et al. 2017), with adaptive intervention protocols informed by real‐time population viability assessments demonstrating documented efficacy in extinction rate mitigation (Lu and Zhang 2013). Comprehensive endangerment profiling integrates demographic stochasticity modeling, biogeographic pattern analysis, and population trajectory forecasting to orchestrate precision conservation initiatives (Fu 1991; Guyennon et al. 2023; Sienkiewicz and Łaska 2023).

The term “endemism” was first described by the botanist Augustin Pyramus de Candolle in 1820, referring to the phenomenon in which a species is exclusively distributed within a specific geographical area (Fouad et al. 2019). Studies have shown that nearly half of the world's vascular plant species and about one‐third of terrestrial vertebrates are endemic to 25 biodiversity hotspots (Brooks et al. 2002). According to the World Checklist of Vascular Plants, a total of 221,399 endemic plant species have been recorded across 173 countries worldwide. China is a key repository of such unique diversity, with 17,700 plant species endemic to the country, accounting for 7.99% of the world's endemic plants (Ministry of Ecology and Environment and Chinese Academy of Sciences 2023; Gallagher et al. 2023).

**FIGURE 1:**
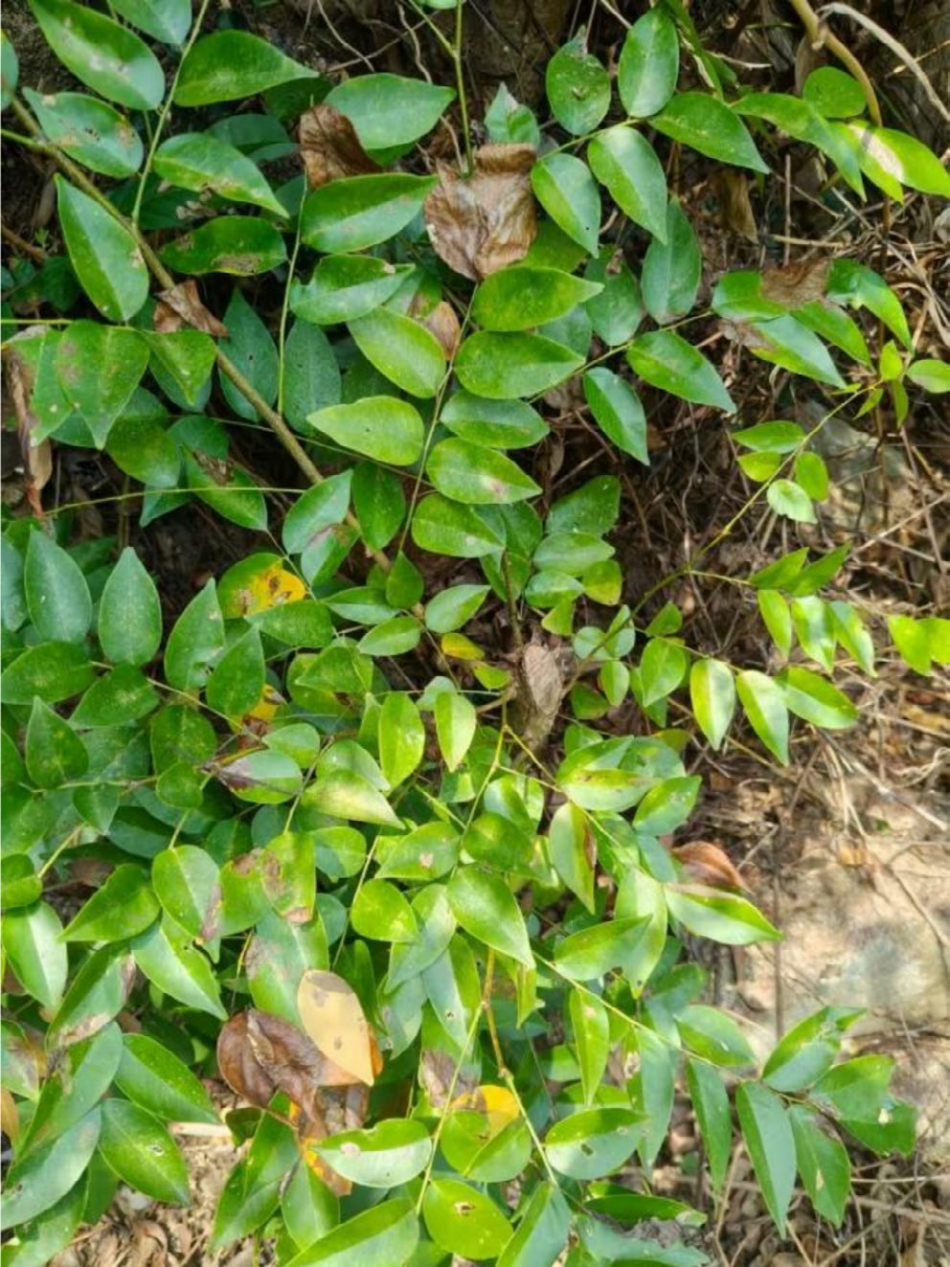
Photograph of *Dalbergia odorifera*.

*Dalbergia odorifera* T.C.Chen is a tree species of the genus *Dalbergia* (Fabaceae family), commonly known as “Fragrant Rosewood” (scientific name: *D. odorifera*) (Figure 1). This species was first described by Merrill and Chen Huan‐yong in 1934, initially encompassing both *D. odorifera* and *D. hainanensis* Merr. & Chun; later in 1963, Chen De‐zhao segregated *D. odorifera* from *D. hainanensis* in the broad sense (Wang et al. 2018). Its wood possesses distinct characteristics: the sapwood is pale yellow with a loose texture, while the heartwood is reddish‐brown with a dense grain. Notably, the heartwood from its roots is the traditional Chinese medicinal material “Jiangxiang”, which exhibits significant analgesic effects and can also be used for treating traumatic bleeding (Editorial Committee of Flora of China 1990). It is native to Hainan Island, China, primarily distributed in open or dense forests on mountain slopes below 700 m elevation in central and southern regions of the island. It is currently listed as a Class II National Protected Wild Plant in China (Editorial Committee of Flora of China, Chinese Academy of Sciences 1990). *D. odorifera* serves not only as a nationally important strategic resource but also as a unique and valuable medicinal herb and aromatic species in China (Zhang et al. 2024). Due to overexploitation and unsustainable human activities, its natural populations have declined drastically (Liu et al. 2019). Current research on *D. odorifera* focuses on stress physiology (Zhou et al. 2025), DNA barcoding for species identification (Tang et al. 2013; Yu 2015), phytochemical compound isolation and characterization (Li et al. 2018), silvicultural and propagation techniques (Lin et al. 2024), and germplasm variation studies (Luo et al. 2022). However, comprehensive investigations into its wild population status and distribution patterns remain lacking. Regarding its conservation status, the 1998 IUCN Red List of Threatened Species classified *D. odorifera* as vulnerable (VU), based on its fragmented wild populations (endemic to Hainan and parts of Southeast Asia) and habitat degradation (SSC/IUCN 2000). In contrast, the 2013 China Biodiversity Red List categorized it as CR. It was further designated as a National Class II Protected Plant in 2021, reflecting the critical endangerment of its domestic wild populations (Ministry of Ecology and Environment and Chinese Academy of Sciences 2023). The discrepancy between domestic (CR) and international (VU) assessments may arise from differences in taxonomic delineation, distribution range criteria, anthropogenic impact evaluations, and assessment methodologies. Recent studies confirm that *D. odorifera* is a distinct endemic species (Wang et al. 2023), suggesting that its international classification as VU underestimates its extinction risk.

Reassessing *D. odorifera* is both scientifically and conservationally critical. Although the species was previously evaluated by the IUCN as VU in 1998—a status that has not been revised since—its actual survival conditions and external environment have changed considerably. In recent years, large‐scale habitat loss and degradation have occurred across its distribution range due to illegal logging, uprooting, deforestation, and agricultural expansion—threats that had not yet emerged or were not fully recognized during the last assessment. Meanwhile, recent systematic field surveys have yielded more comprehensive and accurate data, confirming a sharp decline in the number of mature individuals, the disappearance of multiple historically recorded subpopulations, and the critical endangerment of others. The population decline is far more severe than previously documented, and the quantitative data underpinning the original assessment are no longer sufficient to reflect its current extinction risk.

This study aims to reassess the endangerment status of *D. odorifera* through field surveys of its current wild populations and synthesis of existing literature, thereby providing scientific recommendations for its conservation and management. This reassessment aims to provide timely and reliable evidence to support conservation management, red list updates, and policy formulation, constituting a critical step toward addressing its survival crisis and preventing this unique biological heritage from moving toward extinction.

## 2 Materials and Methods

### 2.1 Overview of the Study Area

Located between 18°10′–20°10′ N and 108°37′–111°03′ E, Hainan Island—the second largest island in China—covers a total area of approximately 33,900 km^2^. The island features a central mountainous region encircled by lower elevations, with its highest peak, Wuzhi Mountain, reaching 1867 m above sea level. It experiences a tropical marine monsoon climate, characterized by year‐round warm and humid conditions, with a mean annual temperature of 22°C–26°C and annual precipitation of 1500–2500 mm. The rainy season from May to October accounts for 70%–90% of the total annual precipitation (Chen et al. 2014). The island's ecosystems exhibit distinct vertical zonation: Latosols are distributed below 300 m, while lateritic soils are found at 300–800 m. The vegetation comprises tropical monsoon forests at lower elevations, transitioning to tropical rainforests (500–1000 m), and further to montane rainforests, evergreen broad‐leaved forests, and summit mossy elfin forests at higher altitudes. As a critical ecotone between the flora of China and Southeast Asia, Hainan Island boasts rich biodiversity, with numerous rare species conserved in the central and southern mountains. However, native vegetation in the northern and coastal areas has been largely converted due to agricultural and urban expansion (Guo et al. 2006; Bosun et al. 2007).

### 2.2 IUCN Red List of Threatened Species and Criteria

The International Union for Conservation of Nature (IUCN), a globally authoritative organization in nature conservation, remains the only international alliance comprising both government and civil society members. Its core conservation tool, the IUCN Red List of Threatened Species, was launched in 1963. As of the 2024‐2 version, the Red List has assessed more than 166,000 species, of which 28% (approximately 46,300 species) are threatened with extinction (IUCN 2024; Forest Focus Network 2024). In China (including Hong Kong, Macao, and Taiwan), a total of 14,520 species have been assessed by the IUCN, with 1968 identified as threatened, providing a fundamental basis for compiling China's Biodiversity Red List (IUCN 2024; Forest Focus Network 2024).

The IUCN Red List of Threatened Species serves as the world's most comprehensive database for documenting the conservation status of global plant and animal species (Rodrigues et al. 2006). The framework of endangerment categories and assessment criteria within this list was initially developed for evaluating species extinction risk at the global scale but is now widely adopted in national‐level conservation efforts (Miller et al. 2007).

**FIGURE 2:**
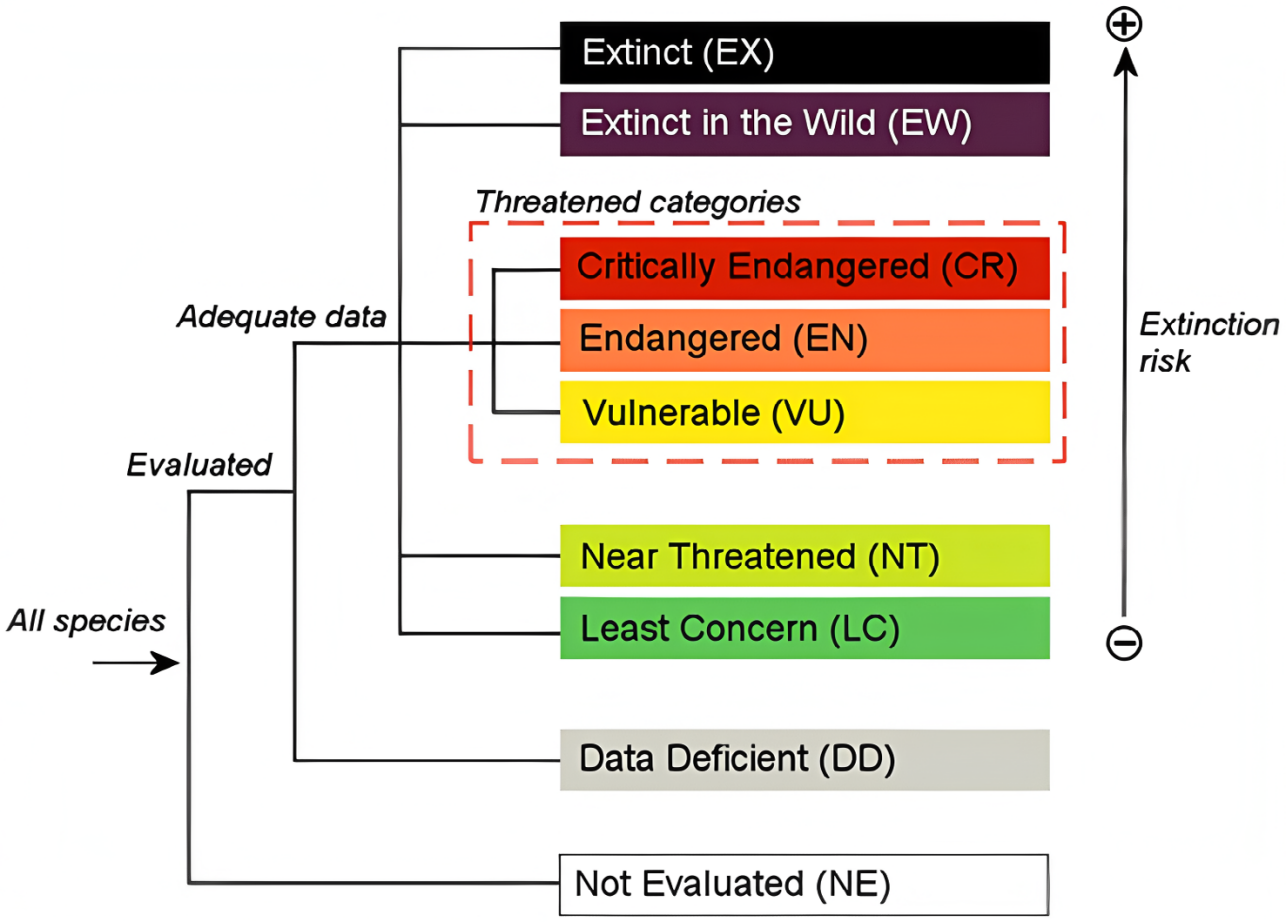
IUCN Red List categories and criteria.

The IUCN Red List Categories and Criteria (Version 3.1), an internationally recognized framework for assessing species endangerment, has served as the global standard for classifying threatened species since its 2001 revision. This system categorizes species into nine distinct risk levels: extinct (EX), extinct in the wild (EW), CR, endangered (EN), VU, near threatened (NT), least concern (LC), data deficient (DD), and not evaluated (NE). CR, EN, and VU are collectively termed “Threatened Categories” (IUCN 2001) (Figure 2). Species assessments rely on five core criteria: (1) Population size reduction dynamics; (2) Current distribution status and decline trends; (3) Number of mature individuals and their trajectory; (4) Geographic fragmentation or small population size; (5) Quantitative predictions of wild extinction probability within 10 years or three generations. While all five criteria are defined, meeting one or more specific sub‐criteria (not necessarily all) is sufficient to assign a threat category (Wang 1996).

### 2.3 Endangerment Assessment System for China's Very Small Populations of Wild Plants

Integrating international principles for plant extinction risk assessment, China's Endangerment Assessment System for Wild Plants with Extremely Small Populations is a scientifically rigorous and widely adopted framework whose selected factors fully account for species' biology, threat responses, and alignment with conservation frameworks (Guo and Zang 2013). Using the analytic hierarchy process (AHP) and combining field surveys with experimental research, this system constructs a hierarchical evaluation model to characterize species' ecological adaptability, supported by consistent calculations and expert consensus.

The system employs a four‐tier structural design: 1 Overall Layer, 2 System Layers, 7 Criteria Layers, and 30 Indicator Layers (Table 1). The formula for calculating indicator scores is:

$$F_{k}=P_{k}/S_{k}$$

Notice:

**TABLE 1:** Evaluation index system and index weights for assessing the endangerment level of wild plants with extremely small populations in China.

Overall layer	System layer		Criteria layer		Indicator layer	
	Index	Weight	Criteria	Weight	Indicator	Weight
Evaluation indicatiors for threatened degree of the tiny population of wild plants endangered in China	Internal indicators	0.5675	Genetic factors	0.2515	Species viability	0.2881
					Species heritability	0.2741
					Distribution frequency	0.2134
					Existing abundance	0.2244
			Reproductive factors	0.2325	Reproduction mode	0.2741
					Reproductive capacity	0.2881
					Population structure	0.2244
					Protective effect	0.2134
			Lifestyle factors	0.2413	Viability	0.2134
					Competitive ability	0.2244
					Population live ability	0.2881
					Individual live ability	0.2741
			Adaption factors	0.2747	Disaster resistance capacity	0.2101
					Drought resistance	0.1854
					Anti‐disturbance	0.1784
					Pest‐resistant	0.2132
					Resistance to industrial pollution	0.2129
	External indicators	0.4325	Human factors	0.2535	Excess deforestion	0.2885
					Grazing	0.2687
					Reclamation	0.2764
					Man‐made fire	0.1664
			Industrial pollution factors	0.4354	Acid rain	0.1224
					Bituminous photochemical smog	0.067
					Pesticide	0.2774
					Greenhouse effect	0.2986
					Pollution of air, water, soil	0.2346
			Natural factors	0.3111	Climate change	0.2881
					Geological disasters	0.1864
					Flood, fire, etc.	0.2546
					Invasive species	0.2709

*F*_*k*_ = indicator evaluation score, quantifying the deviation between the observed value *P*_*k*_ and the standard threshold S_k_;

*P*_*k*_ = empirically measured value derived from field surveys and experimental data;

*S*_*k*_ = safety reference threshold, defined as the benchmark value required to maintain stable wild plant populations.

Building on the Chinese Extremely Small Population Wild Plant Assessment System developed by Guo and Zang (2013), this study conducted an endangerment assessment of *D. odorifera* by systematically scoring its status across 30 quantitative indicators. Scores were assigned based on field survey data of its current wild survival status and documented survival conditions from the literature. For indicators lacking empirical data or literature‐supported values, three experts in botany or ecology were consulted to provide evaluative scores, with the arithmetic mean adopted as the final score for each such indicator (Table 2). A linear weighted average method was used to calculate the comprehensive endangerment score, defined by the formula:

$$C=\sum_{i=1}^{m}\left[\sum_{j=1}^{n}\left(\sum_{k=1}^{j}\left(F_{k}\times P_{k}\right)\times R_{k}\right)\times W_{i}\right]$$

**TABLE 2:** Descriptions and scoring of evaluation indicators.

Index	Factor	Quantitative analysis	Scoring	Normalized value	Evaluation standard
Species viability	Survival rate		1.5	0.5	The highest set of the calculation results is 3:0–1 mean poor species viability; 1–2 mean medium species viability;2–3 mean good species viability
	Premature aging rate	Expert review			
	Healthy rate				
	Mortality rate				
Species heritability	Population inbred rate	Moderate genetic diversity was observed: Nei's gene diversity (0.36), expected heterozygosity (0.37), and observed heterozygosity (0.28). Genetic differentiation among populations was moderate (*F*_st_ = 0.042–0.115), with only 3% of genetic variation existing among populations and 97% within populations Liu et al. (2019).	1.1	0.3667	The highest set of the calculation results is 3:0–1 mean poor species heritability; 1–2 mean medium species heritability; 2–3 mean good species heritability
	Genetic diversity decline rate				
	Genetic drift rate				
Distribution frequency	Species distribution area	Field surveys have currently identified 8 distribution sites with existing wild plants, including 2 in Dongfang, 2 in Sanya, and 4 in Changjiang. All sites support extremely small plant numbers and occupy minimal areas.	4	0.8	The highest set of the investigation factor is 5: 1 mean distribution area is 1; 2mean distribution area is 2–3;3 mean distribution area is 4–6; 4 mean distribution area is 7–10; 5 mean distribution area is 11
Existing abundance	Number of species distribution	Based on current field survey data, fewer than 200 wild *D. odorifera* individuals survive in the wild, with juvenile trees comprising a dominant proportion and mature individuals remaining scarce.	1	0.2	The highest set of the investigation factor is 5: 1 mean the number is from 1 to 500; 2 mean the number is from 501 to 1000; 3 mean the number is from 1001 to 5000; 4 mean the number is from 5001 to 10,000; 5 mean the number is more than 10,000
Reproduction mode	Sexual reproduction rate	The low natural germination rate of *D. odorifera* seeds is a critical issue leading to its endangerment (Wang et al. 2015, Deng 2013).	1	0.333	The highest set of the calculation results is 3: 0–1 mean poor reproduction mode; 1–2 mean medium reproduction mode; 2–3 mean the best reproduction mode
	Asexual reproduction rate				
Reproductive capacity	Selfing rate	Expert review	1	0.333	The highest set of the calculation results is 3:0–1 mean poor reproduction capacity; 1–2 mean medium reproduction capacity; 2–3 mean good reproduction capacity
	Hybridization rate				
Population structure	Life strength	Field surveys revealed that no distribution site maintains a complete population structure; all sites are dominated by juvenile trees, which are extremely low in number.	0.5	0.1667	The highest set is 3: 0–1 mean population structure with poor regeneration capacity and poor stability; 1–2 mean medium population structure with natural regeneration capacity; 2–3 mean population structure with natural regeneration capacity and excellent stability
	Updated force				
	Stability				
Protection effect	Introduction and cultivation survival rate	Seeds used as explants exhibit higher survival rates than stem segments and can develop into complete tissue‐cultured regenerated plants (W. X. Yang 2018). Using shoot tips as explants represents the optimal choice for *D. odorifera* tissue culture Yang et al. (2012).	1	0.3333	The highest set of the calculation results is 3: 0–1 mean poor protection effect; 1–2 mean medium protection effect; 2–3 mean good protection effect
	The seed saved or plant organs, tissues in vitro culture survival rate				
	Reintroduction survival rate				
Viability	Population survivability	Expert review	1	0.333	The highest set of the calculation results is 3: 0–1 mean poor species viability; 1–2 mean medium species viability; 2–3 mean good species viability
	Flowering rate				
	Seed setting rate				
Competition capacity	Competitiveness of the same species	Studies on the natural community of *D. odorifera* have found that the species exhibits a relatively low important value, indicating a weak status and limited functional role within the community.	0.9	0.3	The highest set of the calculation results is 3: 0–1 mean poor competition capacity; 1–2 mean medium competition capacity; 2–3 mean good competition capacity
	Different species of competitive				
Population live capacity	Population survivability	Studies on the natural community of *D. odorifera* have found that the species exhibits a relatively low important value, indicating a weak status and limited functional role within the community.	1.1	0.3667	The highest set of the calculation results is 3: 0–1 mean poor population live capacity; 1–2 mean medium population live capacity; 2–3 mean good population live capacity
	Reproductive rate				
Individual live capacity	Individual survival	The low natural germination rate of *D. odorifera* seeds is a key issue leading to its endangerment; the species can be propagated through asexual reproduction Wang et al. (2015), Deng (2013).	1.3	0.4333	The highest set of the calculation results is 3:0–1 mean poor individual live capacity; 1–2 mean medium individual live capacity; 2–3 mean good individual live capacity
	Individual reproductive rate				
Disaster resistance capacity	Resistance	Expert review	1	0.3333	The highest set is 3: 0–1 mean poor disaster resistance capacity; 1–2 mean medium disaster resistance capacity; 2–3 mean good disaster resistance capacity
Drought resistance	Resistance	Expert review	2	0.6667	The highest set is 3: 0–1 mean poor drought resistance; 1–2 mean medium drought resistance; 2–3 mean good drought resistance
Anti‐disturbance	Resistance	Expert review	1	0.3333	The highest set is 3:0–1 mean poor anti‐disturbance; 1–2 mean medium anti‐disturbance; 2–3 mean good anti‐disturbance
Pest‐resistance	Resistance	Expert review	0.5	0.1667	The highest set is 3: 0–1 mean poor pest‐resistance; 1–2 mean medium pest‐resistance; 2–3 mean good pest‐resistance
Resistance to industrial pollution	Resistance	Expert review	1	0.3333	The highest set is 3: 0–1 mean poor resistance to industrial pollution; 1–2 mean medium resistance to industrial pollution; 2–3 mean good resistance to industrial pollution
Excess deforestion	Vegetation coverage Habitat fragmentation	Due to unsustainable human exploitation and utilization, the natural resources of *D. odorifera* have drastically declined Liu et al. (2019), and wild individuals are highly vulnerable to illegal digging and felling.	0.2	0.0667	The highest set is 3: 0–1 mean low vegetation coverage and high habitat fragmentation; 1–2 mean medium vegetation coverage and habitat fragmentation; 2–3 mean high vegetation coverage and low habitat fragmentation
Grazing	Habitat fragmentation and disappear, habitat fragmentation	Due to unsustainable human exploitation and utilization, the natural resources of *D. odorifera* have drastically declined Liu et al. (2019), and wild individuals are highly vulnerable to illegal digging and felling.	0.2	0.0667	The highest set is 3: 0–1 mean habitat fragmentation is high and habitat fragmentation disappear; 1–2 mean habitat fragmentation is medium and habitat fragmentation disappear; 2–3 mean habitat fragmentation is low and habitat fragmentation disappear
Reclamation	Habitat fragmentation and disappear, habitat fragmentation	Due to unsustainable human exploitation and utilization, the natural resources of *D. odorifera* have drastically declined Liu et al. (2019), and wild individuals are highly vulnerable to illegal digging and felling.	0.2	0.0667	The highest set is 3: 0–1 mean habitat fragmentation is high and habitat fragmentation disappear; 1–2 mean habitat fragmentation is medium and habitat fragmentation disappear; 2–3 mean habitat fragmentation is low and habitat fragmentation disappear
Man‐made fire	Habitat fragmentation and disappear, habitat fragmentation	Due to unsustainable human exploitation and utilization, the natural resources of *D. odorifera* have drastically declined Liu et al. (2019), and wild individuals are highly vulnerable to illegal digging and felling.	0.2	0.0667	The highest set is 3: 0–1 mean habitat fragmentation is high and habitat fragmentation disappear; 1–2 mean habitat fragmentation is medium and habitat fragmentation disappear; 2–3 mean habitat fragmentation is low and habitat fragmentation disappear
Acid rain	PH	Expert review	1	0.3333	The highest set is 3: 0–1 mean acid rain impact on vegetation is poor; 1–2 mean acid rain impact on vegetation is medium; 2–3 mean acid rain impact on vegetation is strong
Bituminous photochemical smog	NMHC/NOX	Expert review	1	0.3333	The highest set is 3: 0–1 mean NMHC/NOX impact on vegetation is poor; 1–2 mean NMHC/NOX impact on vegetation is medium; 2–3 mean NMHC/NOX impact on vegetation is strong
Pesticide	Pesticides concentration	Expert review	1	0.3333	The highest set is 3: 0–1 mean pesticides concentration impact on vegetation is poor; 1–2 mean pesticides concentration impact on vegetation is medium; 2–3 mean pesticides concentration impact on vegetation is strong
Greenhouse effect	Temperature change	Expert review	1	0.3333	The highest set is 3: 0–1 mean temperature change impact on vegetation is poor; 1–2 mean temperature change impact on vegetation is medium; 2–3 mean temperature change impact on vegetation is strong
Pollution of air, water, soil, etc	Pollution index	Expert review	1	0.3333	The highest set is 3: 0–1 mean industry pollution impact on vegetation is poor; 1–2 mean industry pollution impact vegetation is medium; 2–3 mean industry pollution impact on vegetation is strong
Climate change	Climate change	Expert review	1	0.3333	The highest set is 3: 0–1 mean climate change impact on plant germination, leaf unfolding flowering, leaf discoloration is poor; 1–2 mean the impact is medium; 2–3 mean the impact on is strong
Geological disaster	Geological disaster	Expert review	1	0.3333	The highest set is 3: 0–1 mean geological disaster impact on vegetation is poor; 1–2 mean geological disaster impact on vegetation is medium; 2–3 mean geological disaster impact on vegetation is strong
Flood, fire, etc.	Flood, fire indicators	Expert review	1	0.3333	The highest set is 3: 0–1 mean flood, fire impact on vegetation is poor; 1–2 mean flood, fire impact on vegetation is medium; 2–3 mean flood, fire impact on vegetation is strong
Invasive species	Species resistance Alien species invasion	Expert review	1	0.3333	The highest set is 3: 0–1 mean species resistance is poor and species invasive is strong; 1–2 mean both species resistance and species invasive is medium; 2–3 mean species resistance is strong and species invasive is weak

Notice: The comprehensive endangerment index (*C*) for wild plants with extremely small populations is calculated through a three‐tier weighting system comprising m system‐level indicators, *n* criterion‐level indicators, and j indicator‐level metrics;

*F*_*k*_ = indicator evaluation value (defined in Section 2.3);

*P*_*k*_ = weight assigned to the indicator at the criterion level;

*R*_*k*_ = weight of the criterion within its respective criterion layer;

*W*_*i*_ = weight of the system‐level indicator within the overall framework.

Domestic and international standards typically classify EN wild plants into four risk tiers (Wu and Ding 2004): CR (*C* ≤ 0.4), EN (0.4 < *C* ≤ 0.6), rare (0.6 < *C* ≤ 0.8), relatively safe (*C* > 0.8). Building on this framework, Guo and Zang (2013) further refined the classification into four sub‐tiers: level I endangerment (0 ≤ *C* ≤ 0.1), level II Endangerment (0.1 < *C* ≤ 0.2), level III endangerment (0.2 < *C* ≤ 0.3), level IV endangerment (0.3 < *C* ≤ 0.4).

### 2.4 Assessment Steps

To clarify the assessment process for the EN status of *Dalbergia odorifera*, this study adopted a four‐step progressive process to conduct the assessment, with the steps as follows: (1) Preliminary data collection; (2) Field survey verification; (3) Multi‐dimensional assessment; (4) Expert review and confirmation.

First, before conducting formal field surveys, potential distribution areas were screened through the dual channels of literature integration and community interviews. The study systematically collected authoritative resources, including floras such as *Flora of China* (Wu et al. 1994–2013), *Hainan Plant List* (X. B. Yang 2013), *Illustration and Distribution Characteristics of Rare and Protected Plants in Hainan* (X. B. Yang 2017), *Flora of Hainan* (Wu et al. 1964, 1999), and *Flora of Guangdong* (Chen and Huang 2005); *red lists such as Red List of Species in China* (Wang and Xie 2004) and Red List of Chinese Biodiversity—Higher Plants Volume (2020) (Ministry of Ecology and Environment and Chinese Academy of Sciences 2023); as well as published journal articles, specimen records, and historical field survey data (Table 3). It then sorted out basic information about *D. odorifera*, including its historical distribution range, habitat characteristics, and resource change trends. Meanwhile, combined with literature information, the study conducted “non‐induced interviews” with community residents near the survey area who had experience accessing mountainous regions (e.g., shepherds, herbal collectors), asking questions such as whether they had seen this tree species, its distribution location, and recent sighting situations, and marked the potential distribution sites mentioned by the interviewees.

**TABLE 3:** Literature sources overview.

Data category	Name	Author/compiling organization	Time period
Floras	*Flora of China*	Wu Zhengyi, Peter H. Raven Hong Deyuan	1994–2013
Floras	*Hainan Plant List*	Yang Xiaobo	2013
Floras	*Illustration and Distribution Characteristics of Rare and Protected Plants in Hainan*	Yang Xiaobo	2017
Floras	*Flora of Hainan*	Wu Deling	1964; 1999
Floras	*Flora of Guangdong*	Chen Huanyong	2005
Red lists	*Red List of Species in China*	Wang & Xie	2004
Red lists	*Red List of Chinese Biodiversity—Higher Plants Volume (2020)*	Ministry of Ecology and Environment & Chinese Academy of Sciences	2023
Additional data	Published journal articles	Liu et al. (2019), Zhou et al. (2025), Tang et al. (2013), Lin et al. (2024), Wang et al. (2015), Deng (2013), Yang et al. (2012).	—
Additional data	Specimen records	Chinese Virtual Herbarium(CVH); National Plant Specimen Resource Center(NPSRC)	—
Additional data	Field survey data	The author's affiliated team	2020–2024

Second, based on the potential areas marked during the interviews, the research team was guided by interviewees to the suspected distribution sites and adopted the typical plot method to conduct field surveys. A total of 15 plots (each 20 m × 20 m) were established in the marked areas. The study recorded the number of *D. odorifera* plants, diameter at breast height (DBH) or diameter at ground level, and health status within each plot, as well as types of habitat disturbances in the plots (e.g., logging traces, grazing activities).

Third, the study combined international and domestic evaluation systems for plant species with extremely small populations to conduct a multi‐standard integrated assessment of the EN status. On one hand, it strictly followed the IUCN Red List categories and criteria (Version 3.1) and regional application guidelines, focusing on analyzing core indicators such as population size (Criterion A), geographical distribution (Criterion B), population decline (Criterion C), very small or restricted population (Criterion D), and quantitative analysis (Criterion E). On the other hand, it incorporated China's Endangerment Evaluation System for Wild Plants with Extremely Small Populations, and set 30 assessment indicators (Table 3) from multiple internal and external dimensions, including viability, genetic capacity, distribution frequency, existing abundance, reproductive mode, population structure, habitat adaptation, human disturbance, and pollution. After scoring each indicator, normalization (original score/maximum score) was performed, followed by a linear weighted average calculation.

Finally, the study invited 3 experts in the fields of plant taxonomy, ecology, and conservation biology (including a scholar with IUCN assessment experience). Through a special symposium, the experts reviewed each link one by one, such as the authenticity of distribution data, the rationality of indicator weights, and the degree of standard matching. They also conducted cross‐validation in combination with existing conservation levels (e.g., those in the Red List of Chinese Biodiversity—Higher Plants Volume (2020)), and finally confirmed the EN status of *D. odorifera*.

## 3 Results

**FIGURE 3:**
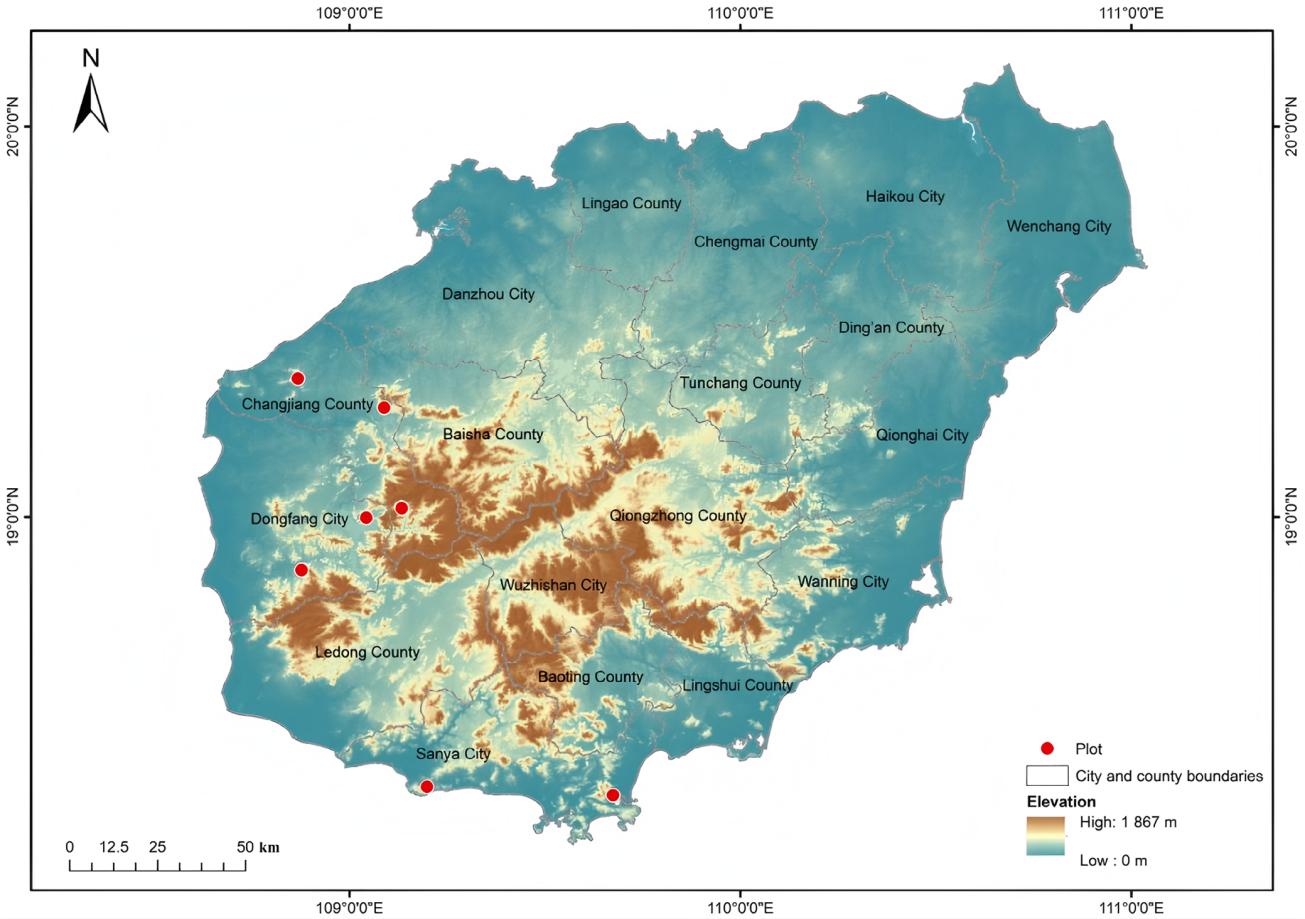
Distribution map of *Dalbergia odorifera*. (Two points in Changjiang appear to overlap at this scale in figure.)

Field surveys reveal critically limited wild distribution of *D. odorifera*, with only eight documented populations in Dongfang (2 sites), Sanya (2 sites), and Changjiang (4 sites) (Figure 3). These populations exhibit alarmingly low densities—individual sites often contain single surviving plants, primarily root sprouts from poached mature trees. Occupying < 5% of community areas, these fragmented habitats show severe anthropogenic degradation.

**FIGURE 4:**
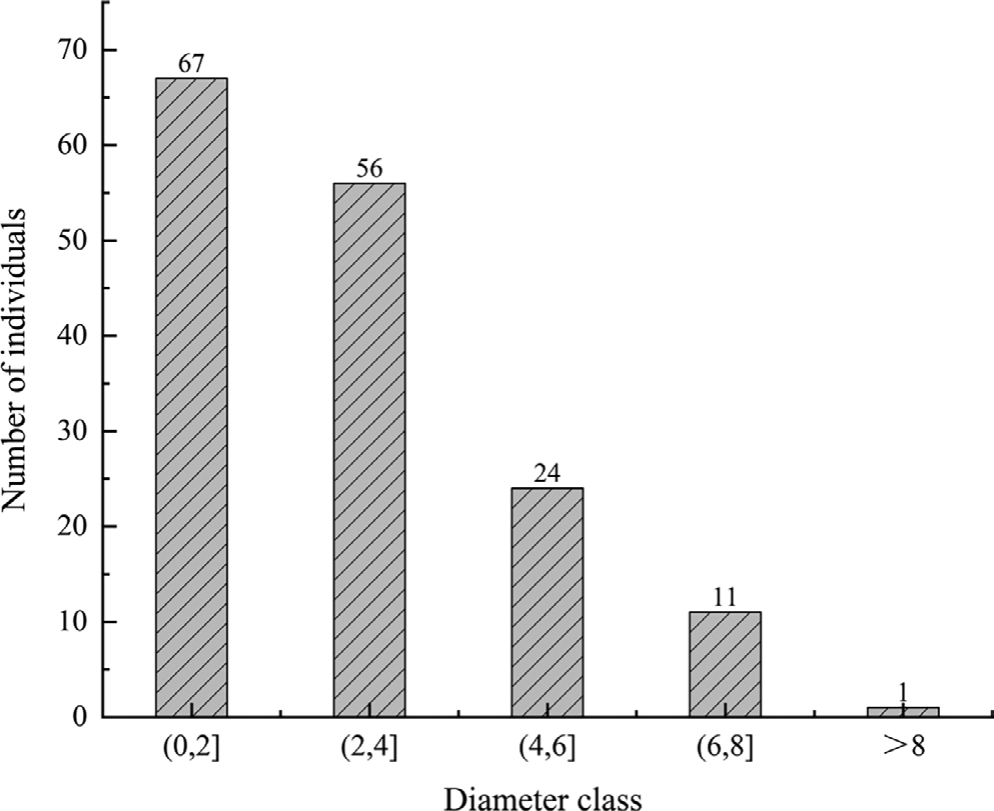
The age class structure and quantity of the wild population of *Dalbergia odorifera*.

**FIGURE 5:**
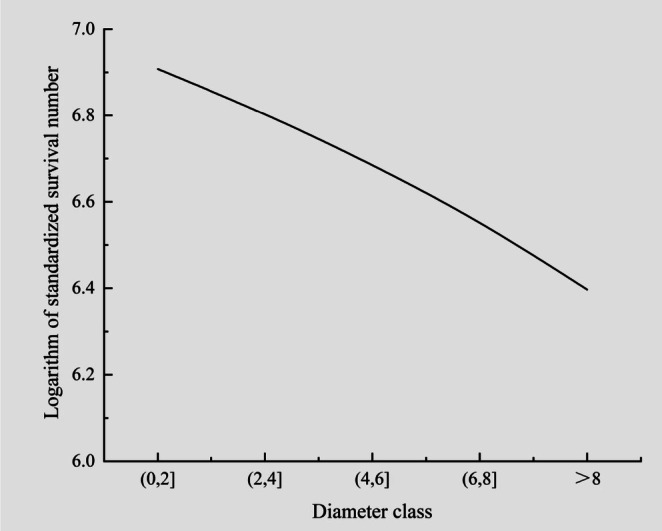
Survival curve of wild population of *Dalbergia odorifera*.

Population structure analysis (space‐for‐time substitution method) using 2‐cm diameter‐at‐breast‐height (DBH) intervals indicates the dominance of juvenile trees, with mature individuals (DBH ≥ 5 cm) constituting merely 20% (≈20 plants) (Figure 4). The derived survival curve demonstrates significant negative skewness (log‐transformed standardized survival vs. DBH class), reflecting progressive mortality with ontogenetic development (Figure 5). Illegal harvesting targeting mature trees for high‐value heartwood has precipitated demographic collapse, exacerbated by extreme spatial segregation among populations (“island” fragmentation pattern). Based on an analysis of the five core criteria in the IUCN Red List Categories and Criteria (Version 3.1): (1) Criterion A (Population Size Reduction) meets Subcriterion A1ac (A. Population size has been reduced by any of the following: 1. An observed, estimated, inferred or suspected reduction of at least 90% over the past 10 years or three generations (whichever is longer), where the causes of reduction are clearly reversible and understood, and have ceased, based on (and specifying) any of the following: a. Direct observation; c. A decline in area of occupancy, extent of occurrence and/or quality of habitat); (2) Criterion D infers that the number of mature individuals in the population is less than 50. Although the fragmentation of its distribution area is also observed, it is not the primary threat factor for the species. Additionally, due to the lack of long‐term population monitoring data, a standard quantitative analysis could not be conducted. Therefore, the wild survival status of *D. odorifera* conforms to CR A1ac; D in the IUCN Red List Categories and Criteria, i.e., it is classified as CR.

This study implemented a conservation priority assessment for *D. odorifera* through China's Wild Plants with Extremely Small Populations (WPESP) Risk Evaluation Framework. The hierarchical assessment matrix incorporated endogenous vulnerability factors (genetic diversity, reproductive strategies, life history traits, adaptive capacity) and exogenous threat metrics (anthropogenic disturbances, industrial pollution intensity). All indicators underwent min‐max normalization (normalized value = raw score/maximum possible score) to standardize measurement scales, thereby mitigating metric inflation risks caused by disproportionate weighting of individual parameters.

The normalized scores were integrated via linear weighted aggregation: Composite Endangerment Index *C* = 0.3253. Cross‐referencing with the IUCN‐CMP Unified Threat Classification System and China's State Council Decree No. 32 conservation taxonomy, *D. odorifera* was classified as EN with Threat Level IV (0.3 < *C* ≤ 0.4). This determination aligns with domestic CR designation under GB/T 2828–2022 standards. We recommend maintaining the current protection status given consensus across assessment protocols.

## 4 Discussion

A systematic assessment based on the criteria of the International Union for Conservation of Nature (IUCN) Red List shows that plant diversity in multiple key regions worldwide is facing severe threats. In the Hawaiian Islands, a preliminary assessment of all 1044 native vascular plant species indicates that as many as 72% (753 taxa) are in a threatened status; among these, 5% of the 256 single‐island endemic vascular plant species on Kauai have gone extinct, representing the highest extinction risk recorded to date (Rønsted et al. 2022). Similarly, a comprehensive tree species assessment in the Atlantic Forest biodiversity hotspot—conducted via automated analysis—rediscovered 5 tree species previously listed as “Extinct” and confirmed that 13 endemic species may have disappeared (de Lima et al. 2024). In Greece, an assessment based on detailed distribution data and a phylogenetic framework shows that most endemic plant taxa in the country face survival threats; among these, 14 evolutionarily distinct and globally EN endemic species require priority conservation (Kougioumoutzis et al. 2021). Furthermore, Litsea quercifolia (an Indonesian endemic species with an extremely narrow distribution range, commonly known as “oak‐leaf litsea”) has not been recorded since its first collection in 184 and has been listed as a CR species. This indicates that some rare taxa may remain in an overlooked EN status for an extended period (Primananda et al. 2023).

Field resources, including the number of individual plants, populations, normal reproductive capacity, and distribution area, are used to assess the EN status of this species (Yang et al. 2019; Liu et al. 2021). In recent years, Chinese scholars have conducted extensive studies on wild plant endangerment assessment. For example, Liu et al. (2021) studied *Diospyros sutchuensis*, which was classified as EN due to scarce field resources, clustered distribution points, and vulnerability to excavation and habitat destruction. In contrast, *D. odorifera* faces endangerment primarily from its high economic value, with habitat destruction caused by agricultural expansion exacerbating threats. A study reported approximately 1100 individual plants of *Michelia guangdongensis* across four wild distribution sites in Guangdong nature reserves, leading researchers to classify it as EN (Yang et al. 2019). Conversely, Li et al. (2017) found that *M. guangdongensis* has only four distribution sites with fewer than 50 mature individuals and subpopulations, qualifying it as CR under IUCN criteria (CR C2a(i); D). In a similar analysis of Jinsha River endemic plants, Yu et al. (2024) determined that *Hibiscus drylandii* (dryland hibiscus) had nearly 11 distribution habitats but suffered severe isolation, small population size, and fewer than 1500 total individuals, resulting in an EN rating.

This study, based on systematic collation of historical distribution data and field transect surveys, found that *D. odorifera* has only 8 known wild distribution points, each with extremely small plant numbers—some sites contain only a single individual, often a small root sprout surviving after adult plants were poached, making them highly susceptible to death. Fewer than 200 wild *D. odorifera* plants exist, with young trees dominating and only ~30 mature individuals. According to IUCN criteria CR A1ac; D, this study classifies it as CR. Notably, many remaining plants grow outside protected areas, and larger individuals have already suffered significant damage. *D.odorifera* is classified as VU in the IUCN Red List of Threatened Species but CR in China's Biodiversity Red List: Higher Plants Volume (2020) (Ministry of Ecology and Environment and Chinese Academy of Sciences 2023). Discrepancies between domestic and international assessments arise from differences in taxonomic definitions, distribution range delineation, intensity of anthropogenic interference, and assessment criteria. Domestically, *D. odorifera* is recognized as an endemic CR species, emphasizing the extreme peril of its wild populations; the international assessment may conflate it with a species complex and include Southeast Asian populations, thereby underestimating its endangerment level—a conclusion that contradicts on‐the‐ground realities and fails to reflect its true survival status. To clarify the causes of this dire state, *D. odorifera* faces four key threats that drive its endangerment: (1) Illegal logging: Its heartwood, as a high‐value mahogany raw material with strong market demand, has led to rampant poaching of mature individuals (with a diameter at breast height (DBH) ≥ 5 cm), even complete uprooting, resulting in a sharp decline in the number of wild adult populations; (2) Habitat destruction and fragmentation: Agricultural expansion and infrastructure construction have caused a drastic shrinkage in the area of its natural habitat (mainly tropical monsoon forests in Hainan), fragmenting the remaining populations into 8 isolated “island‐like” distribution sites (2 in Dongfang City, 2 in Sanya City, 4 in Changjiang County). Some distribution sites have only 1–2 individuals, most of which are seedlings sprouted from residual lateral roots after adult plants were poached and dug up; (3) Restricted natural reproduction: The reproductive rate of populations has decreased after the poaching of adult individuals. During the survey, it was found that the crowns of some adult plants have been covered by vines, with branches and leaves withered, which seriously affects their flowering and fruiting. Ma et al. (2017) applied both IUCN standards and China's extremely small population endangerment assessment system to evaluate *Scutellaria tsinyunensis*, achieving consistent results under both frameworks. Similarly, this study's dual assessment of *D. odorifera* using these two standards also yielded CR, underscoring the dire state of its wild populations. Conducting regional endangerment assessments in China can provide granular data on population distribution and size, laying a robust foundation for scientific evaluations (Lu and Zhang 2013). *D. odorifera* is not only of immense economic value but also embodies unique regional and cultural identities. Its conservation transcends mere species rescue; it constitutes a comprehensive effort to safeguard ecological security, preserve cultural heritage, promote species sustainability, and fulfill international conservation commitments. Its significance far exceeds monetary value, reflecting the profound philosophy of harmonious coexistence between humanity and nature.

## 5 Protection Proposals

To effectively conserve *D. odorifera*—a species of exceptional economic, regional, and cultural value—the following protection recommendations are proposed:

### 5.1 Law Enforcement and Public Engagement

#### 5.1.1 Strengthen Regulatory Enforcement

Enhance oversight of wild *D. odorifera* trade by establishing robust transaction registration systems to ensure traceability of legal origins and destinations. Strictly combat illegal logging and trade through regular special enforcement campaigns by forestry authorities, with severe penalties for violators and associated stakeholders in accordance with the law.

#### 5.1.2 Restrict Harvesting

Impose strict limits on wild *D. odorifera* felling, prohibiting all forms of harvesting except for scientific research or exceptional circumstances approved by relevant authorities. Rigorously review felling permits to ensure compliance with legal and regulatory standards.

#### 5.1.3 Establish a Reporting Mechanism

Encourage public reporting of illegal logging and trade through dedicated hotlines, with strict protection of informant identities and material incentives for valid reports.

#### 5.1.4 Public Education and Outreach

Leverage media and community campaigns to educate the public on the ecological significance, cultural value, and legal frameworks for *D. odorifera* conservation. Organize protection‐themed activities to foster public awareness and cultivate a societal culture of collective conservation.

#### 5.1.5 Limit Non‐Essential Use

Restrict *D. odorifera* timber applications to non‐essential sectors, prioritizing its use in cultural heritage preservation and scientific research. Promote the development of alternative materials to reduce dependency on this species.

### 5.2 Habitat Protection and Restoration

#### 5.2.1 Scientific Reserve Designation

Establish nature reserves in areas with concentrated wild populations, clearly demarcating boundaries and enhancing management. For populations outside reserves, create in situ conservation sites. Conduct comprehensive ecological restoration around reserves to minimize anthropogenic disturbances.

#### 5.2.2 Ecological Rehabilitation

Restore fragmented habitats of *D. odorifera* through reforestation, vegetation restoration, and soil improvement measures to enhance ecosystem stability and connectivity.

### 5.3 Scientific Research and Monitoring

#### 5.3.1 Targeted Multidisciplinary Research

Integrate insights from population ecology, conservation genetics, and reproductive biology to advance applied conservation strategies. Prioritize studies on wild population dynamics, community characteristics, and habitat requirements to inform wild reintroduction and ecological restoration. Use genetic analyses to guide seed resource protection and determine sampling sizes for ex‐situ conservation and propagation.

#### 5.3.2 Long‐Term Systematic Monitoring

Develop a standardized monitoring framework to track distribution patterns, population trends, and demographic changes in wild *D. odorifera*. Base conservation decisions on long‐term data collection across growth, reproduction, and population viability metrics to enable adaptive management.

### 5.4 Interagency Coordination and Policy Support

#### 5.4.1 Cross‐Sector Collaboration

Launch a centralized data‐sharing platform to aggregate multisource data from research institutions and conservation agencies on in situ and ex‐situ conservation efforts, facilitating a holistic understanding of species status.

#### 5.4.2 Policy Incentives for Cultivation

Governments should introduce supportive policies to encourage commercial cultivation of *D. odorifera*, including tax incentives and financial subsidies for growers to promote sustainable industrial development.

#### 5.4.3 International Cooperation

Engage with global conservation organizations and foreign counterparts to share best practices and collaborative research, leveraging international expertise to advance wild population protection aligned with IUCN and domestic standards.

## 6 Conclusion

It is hoped that government agencies will prioritize these evidence‐based recommendations to safeguard *D. odorifera*—an irreplaceable species critical to ecological integrity, cultural heritage, and global conservation commitments.

## Author Contributions

**Chumin Ye:** data curation (lead), formal analysis (lead), investigation (lead), methodology (lead), software (lead), visualization (lead), writing – original draft (lead), writing – review and editing (lead). **Kai Zhang:** data curation (equal), formal analysis (equal), investigation (equal), methodology (equal), software (equal), writing – review and editing (equal). **Xinli Gui:** data curation (equal), investigation (equal), writing – review and editing (equal). **Yukai Chen:** conceptualization (lead), funding acquisition (lead), methodology (equal), writing – review and editing (equal). **Haifu Meng:** investigation (equal), writing – review and editing (equal).

## Conflicts of Interest

The authors declare no conflicts of interest.

## Data Availability

The data used in this study can be found at https://figshare.com/s/8242861491caa728bae9.
